# An Elderly Man with Syncope, Hypoxia, and Confusion: A Case Report and Review of Literature

**DOI:** 10.7759/cureus.5567

**Published:** 2019-09-04

**Authors:** Venu Madhav Konala, Srikanth Naramala, Sreedhar Adapa, Narothama Reddy Aeddula, Subhasish Bose

**Affiliations:** 1 Internal Medicine / Hematology and Oncology, Ashland Bellefonte Cancer Center, Ashland, USA; 2 Rheumatology, Adventist Medical Center, Hanford, USA; 3 Nephrology, The Nephrology Group, Visalia, USA; 4 Medicine and Nephrology, Deaconess Health System / Indiana University School of Medicine, Evansville, USA; 5 Nephrology / Internal Medicine, Lynchburg General Hospital, Lynchburg, USA

**Keywords:** thrombolytic therapy, anticoagulation, pulmonary embolism, patent foramen ovale, paradoxical embolism

## Abstract

Controversies exist regarding the treatment of acute massive pulmonary embolism (PE) with anticoagulation alone or with thrombolytic therapy. Paradoxical embolism in the presence of a patent foramen ovale (PFO) is a rare but well-known entity and should always be looked for in case of a PE associated with systemic thromboembolism. We report a case of acute sub-massive PE treated with thrombolytic therapy in an elderly gentleman who had a paradoxical embolism and ischemic stroke as a result of a clot traversing through a PFO. We discussed diagnostic modalities, treatment of choice, and associated controversies in management.

## Introduction

Paradoxical embolism associated with patent foramen ovale (PFO) is a rare entity. It should always be considered in a patient with pulmonary embolism (PE) associated with systemic thromboembolism. The risk of stroke is four times higher in patients with PFO as compared to patients without PFO [1]. Contrast transesophageal echocardiogram (TEE) was more sensitive than contrast transthoracic echocardiogram (TTE) in identifying PFO [2]. Two major therapeutic options to consider for a patient with right atrial thrombus in the setting of PE are thrombolysis and surgical thrombectomy. The optimal management of patients with paradoxical embolism from PFO is challenging. It must be individualized based on the potential benefits and risks considering the patients' comorbidities along with procedural complications [3]. We report an elderly patient who presented with an acute sub-massive PE treated with thrombolytic therapy and had a paradoxical embolism and ischemic stroke as a result of a clot traversing through a PFO.

## Case presentation

An 82-year-old male with a history of type 2 diabetes mellitus and previous lower extremity deep venous thrombosis six years ago (treated for six months with warfarin) was brought to the emergency department (ED) following an episode of loss of consciousness. The episode occurred soon after he urinated in the bathroom at home and lasted for about two minutes. He felt light-headed and had mild dyspnea before syncope but denied chest pain, palpitations, or any head trauma. He denied recent leg edema.

**Figure 1:**
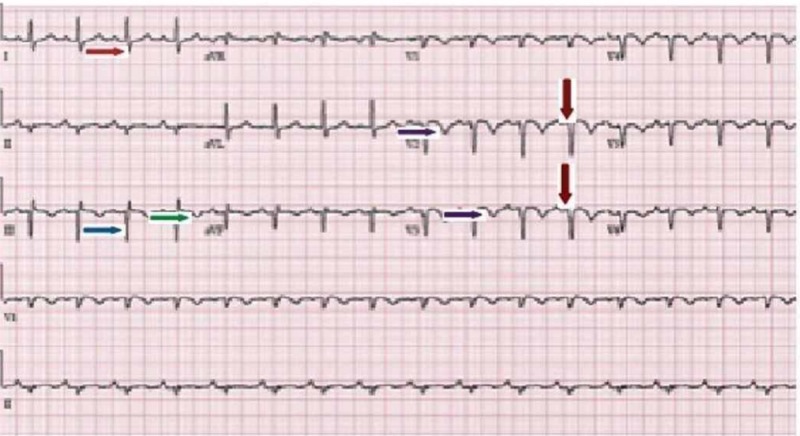
Electrocardiogram (EKG) demonstrated normal sinus rhythm with Q waves (blue arrow) in the inferior leads (II, III, and aVF), a prominent S wave (red arrow) in the lead I, and T wave inversion (green arrow) in the lead III. Diffuse T wave inversions (purple arrows) and poor R wave progression (brown arrows) were noted in anterior precordial leads.

**Figure 2:**
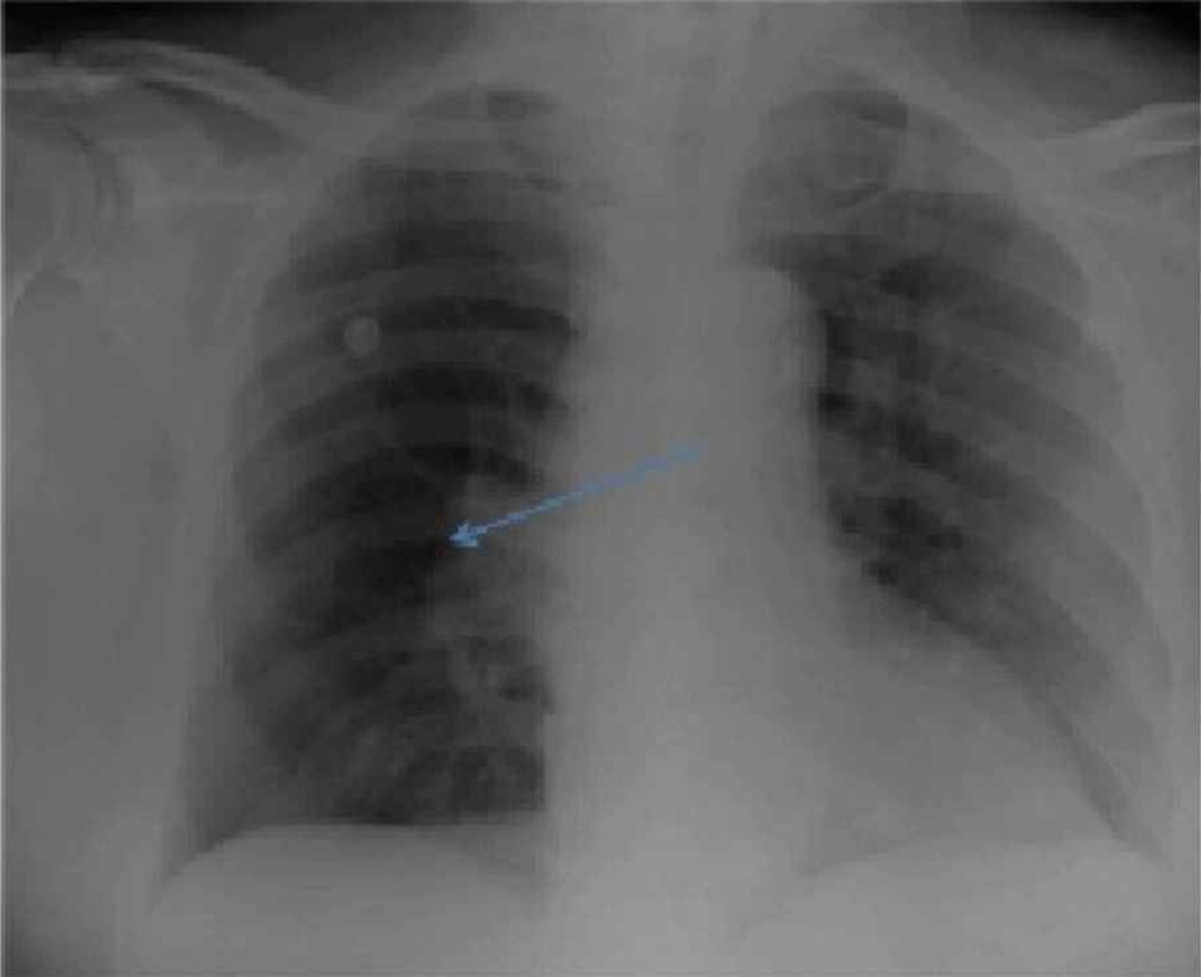
Initial portable anteroposterior chest X-ray (CXR) showing right-sided hilar vascular prominence for dilated pulmonary artery (Fleischner sign) and lung field oligemia, especially evident on the right side (Westermark sign)along with an abrupt absence of distal vessels.

**Figure 3:**
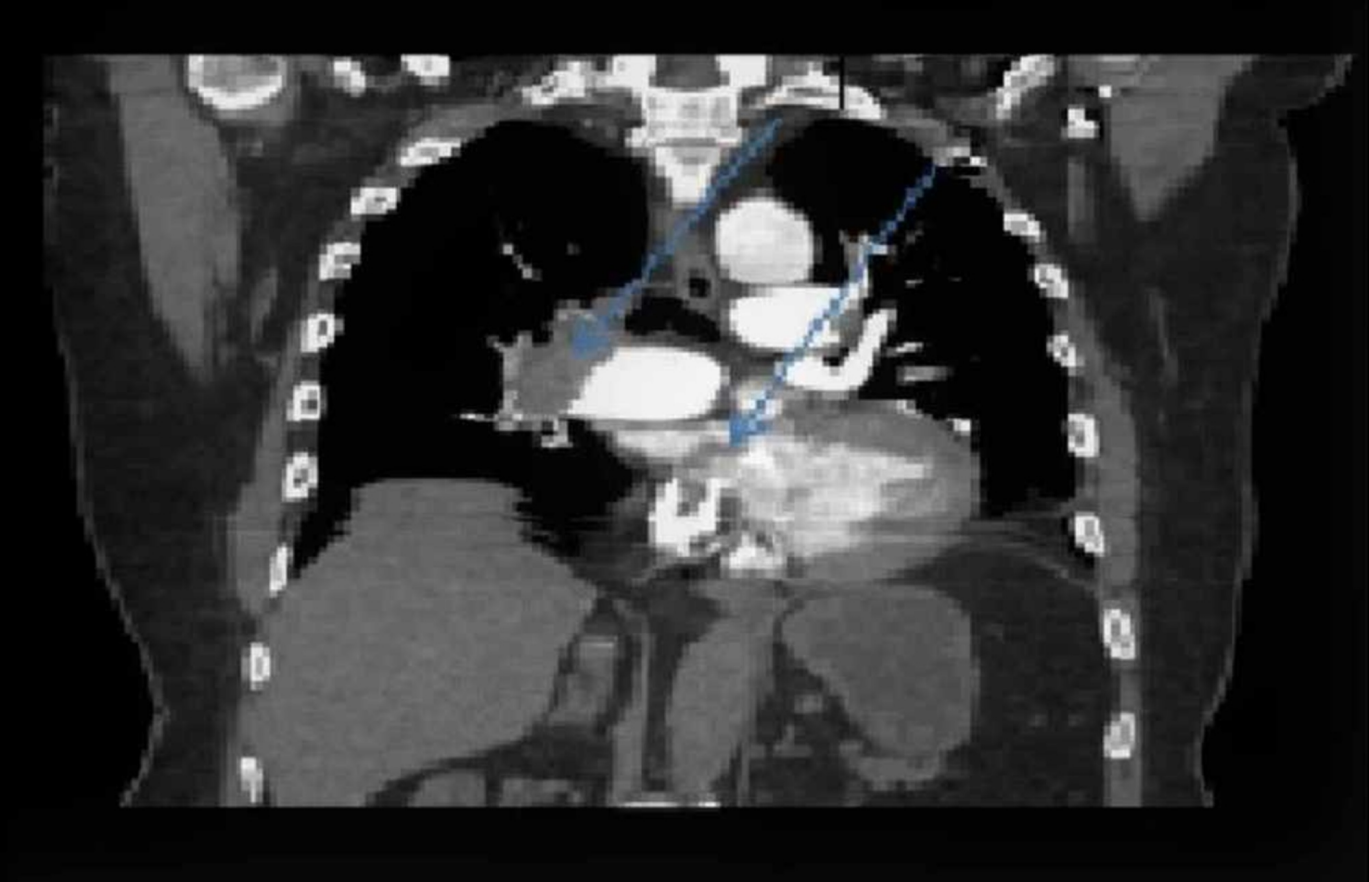
Computed tomography (CT) scan of the chest with intravenous (IV) contrast, coronal section. 1. Right main pulmonary artery occluded with thrombus. 2. A clot is clearly visible to be traversing from the right atrium to the left through the patent foramen ovale (PFO).

On arrival in the ED, his vital signs included a heart rate of 113 beats per minute, blood pressure of 128/69 mmHg, a respiratory rate of 24 breaths per minute, and oxygen saturation of 86% on room air. He was oriented to time, place, and person. His chest was clear to auscultation, and the cardiovascular exam revealed a holosystolic grade 2/6 murmur at the left lower sternal border. His abdomen examination was benign, and there was no lower extremity edema. Electrocardiogram (EKG) demonstrated normal sinus rhythm with Q waves in the inferior leads (II, III, and aVF), a prominent S wave in lead I, and T wave inversion in lead III. Diffuse T wave inversions and poor R wave progression were noted in the anterior precordial leads (Figure 1). No prior EKGs were available for comparison. Complete blood count was within normal limits. The basic metabolic panel was significant for a serum creatinine of 1.5 mg/dl. Troponin I was elevated at 1.5 ng/ml (normal range 0.00 - 0.05 ng/ml). A chest X-ray (CXR) showed right hilar vascular prominence along with an abrupt absence of distal vessels (Figure 2). The cardiac silhouette was normal. Urgent computed tomography (CT) of the chest with intravenous contrast showed extensive pulmonary emboli within the pulmonary arterial tree with near occlusive thrombus in the right main pulmonary artery and nonocclusive thrombus in the left main pulmonary artery. There was a clot seen in the right atria, traversing through a patent foramen ovale (PFO) into the left atria (Figure 3). The right ventricle appeared enlarged with a flattening of the interventricular septum, consistent with severe right heart strain.

**Figure 4:**
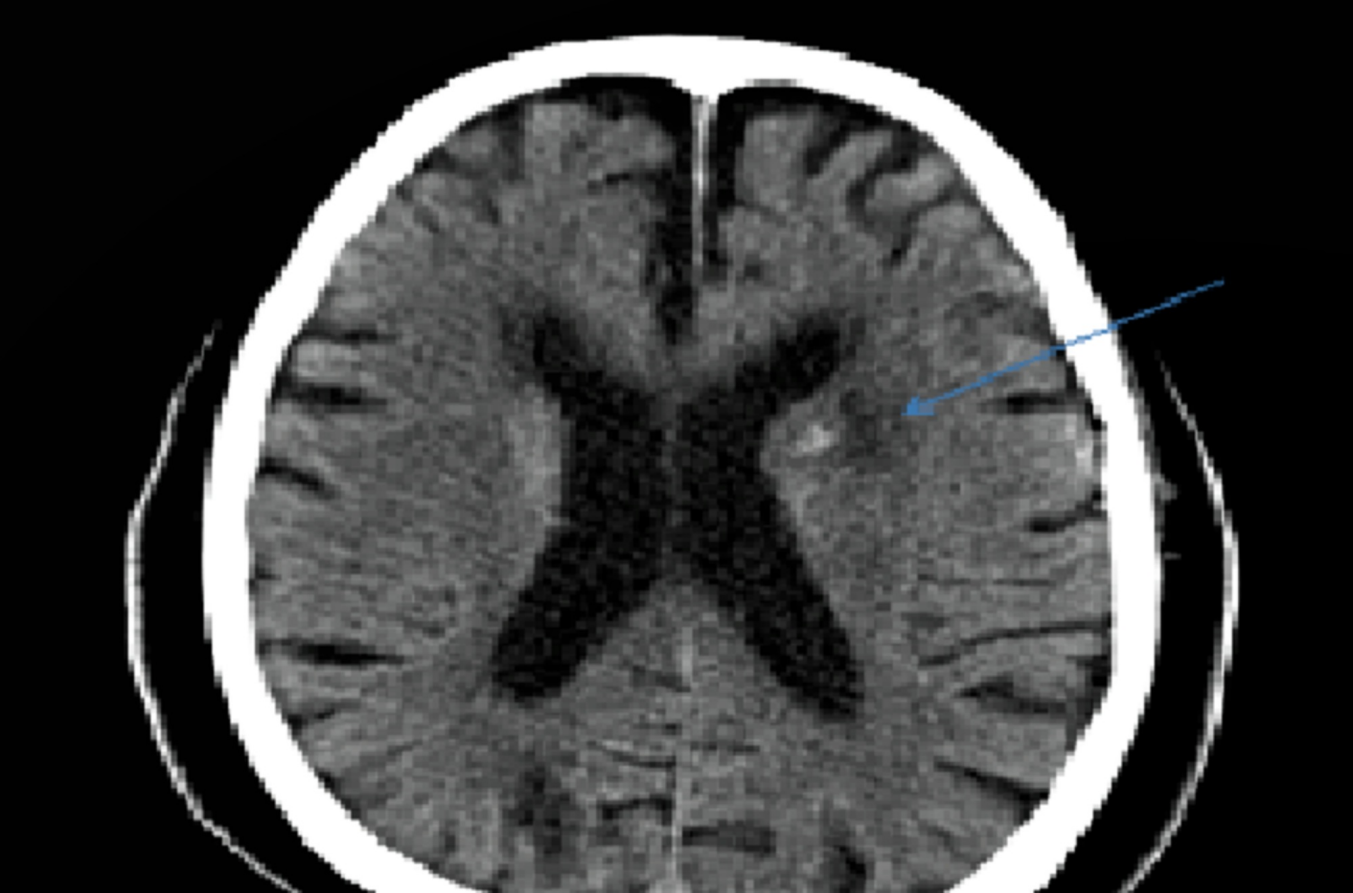
Computed tomography (CT) head without contrast showing acute ischemic infarction in the left caudate/putamen area.

**Figure 5:**
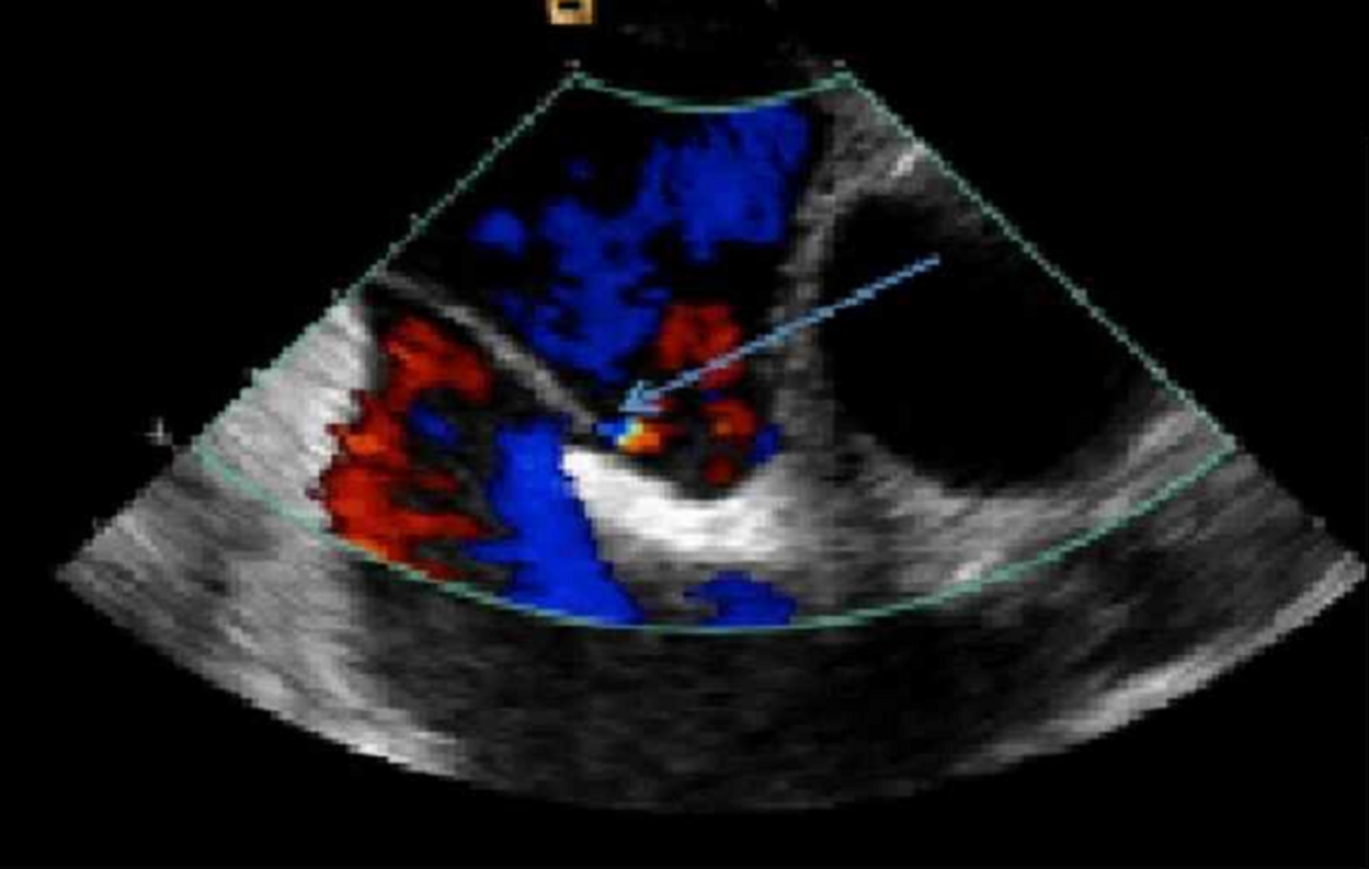
Transesophageal echocardiogram (TEE) with color Doppler showing a patent foramen ovale (PFO) with a right to left shunt

In light of the thrombus visualized in the right and left atria, massive clot burden, worsening hypoxia, and presence of a PFO, the decision was made to administer thrombolytic therapy with alteplase (100 mg over 2 hrs) followed by continuous intravenous heparin infusion with a partial thromboplastin time (PTT) goal of 60 to 80 seconds. The patient’s oxygenation improved significantly, with an oxygen saturation of 96% on 2L per minute oxygen via nasal cannula. Five hours after thrombolytic infusion, he developed an acute onset of confusion. A non-contrast head CT showed acute ischemic infarction in the left caudate/putamen area with hemorrhagic conversion and focal hypodensities in the posterior limb of the right internal capsule/thalamus representing old lacunar infarcts (Figure 4). Doppler ultrasound of the lower extremities showed bilateral partially occlusive superficial femoral and popliteal deep venous thromboses. A transthoracic echocardiogram (TTE) performed one day after thrombolytic therapy showed a dilated right ventricle with severely reduced right ventricle (RV) systolic function. No thrombus was visualized on TTE. A transesophageal echocardiogram (TEE) two days post-thrombolysis revealed a patent foramen ovale with a right to left shunting (Figure 5), and layered, organized thrombus on the wall of the left atrium adjacent to the interatrial septum. The patient underwent the placement of an inferior vena cava filter and was continued on warfarin therapy. Percutaneous closure of the PFO was recommended: however, the patient refused. He was discharged home in stable condition on warfarin therapy.

## Discussion

The mechanism of paradoxical embolism through a PFO was first described in 1877 by Cohnheim. There is a higher risk of cerebral and peripheral ischemic events due to paradoxical embolism from PFO. Acute pulmonary embolism causes elevated back pressure in the pulmonary vasculature, leading to a right-to-left shunt through the PFO, which is associated with high morbidity and mortality [4]. Le Moigne et al. presented the most extensive prospective study to date in patients with acute PE, identifying the presence of ischemic stroke and PFO, with results confirming the presence of ischemic stroke in 21.4% and 5.5% with and without PFO, respectively. This study confirms that PFO and aneurysmatic atrial septum are risk factors for stroke [1].

The EKG is abnormal in over two-thirds of patients with PE. Sinus tachycardia is the most frequent EKG abnormality but lacks specificity. Our patient's EKG showed a prominent S wave in lead I and a Q wave along with T wave inversion in lead III. This classic S1Q3T3 pattern represents right ventricular strain from a large PE. The anterior and inferior T-wave inversions are the most common EKG findings due to massive and submassive PE, as in our patient. Even though the sensitivity and specificity of EKG findings in the diagnosis of PE are low, the resolution of the EKG findings may have prognostic implications and, hence, it is recommended to obtain serial EKGs in this group of patients [5].

On CXR, the finding of hilar vascular prominence with an abrupt absence of distal vessels is known as the Westermark sign (as seen in the CXR of our patient). This finding is uncommon in patients with PE but has high specificity [6].

Transesophageal echocardiography (TEE) can demonstrate intracardiac thrombus, and its utility to diagnose acute pulmonary embolism has been assessed in many studies [7-8]. TEE, in contrast to transthoracic echocardiography (TTE), provides direct visualization of the thrombus in the central pulmonary artery, as compared to the indirect signs seen with TTE [9]. In a study done by Doyen et al., on 41 patients with intermediate-risk PE, contrast TEE was more sensitive than contrast TTE in identifying PFO, with 56% vs. 19.5%, respectively. Ischemic stroke was detected in approximately 17% of the patients in this study, and a PFO with a large shunt was associated with it [2]. Direct visualization of proximal pulmonary arterial thrombi by TEE is emerging as a useful method for the prompt diagnosis of hemodynamically significant pulmonary emboli [9]. The need for sedation makes TEE less readily available in an acute setting such as the emergency room. Chest CT with IV contrast, on the other hand, is readily available as a noninvasive and quick diagnostic modality with excellent sensitivity. In a study by Pruszczyk et al., PE was confirmed by a high-probability lung scan or angiography in 40 patients. A total of 32 (80%) and 36 (90%) patients had central pulmonary arterial emboli identified on TEE and spiral CT, respectively. The specificity is 100% with no false central PE reported by either method [10]. In another study involving 44 patients with clinically suspected acute pulmonary embolism, the results of helical CT or contrast angiography were compared to TEE; TEE had limited accuracy for detecting pulmonary embolism with acute cor pulmonale. TEE was able to confirm the diagnosis of PE in the main or right pulmonary artery within a few minutes, without the need for further invasive testing; however, it did not exclude left proximal or lobar pulmonary embolism in patients with negative TEE [11]. In our patient, chest CT not only demonstrated extensive clots in the pulmonary tree but also revealed an atrial clot, a clot within the interatrial septum, and significant right ventricle (RV) strain.

Thrombus in the right heart is more frequently located in the right atrium (RA) than in the RV and is a potential source of pulmonary embolism [12]. The thrombus is formed in situ due to stasis, which could be due to decompensated congenital or acquired cardiac disease or due to the presence of a foreign body such as a pacing wire. The thrombus is usually non-mobile and adherent. It is either implanted on the free wall of the right atrium or the interatrial septum. It is less likely to cause a pulmonary embolism. It decreases in size or disappears with anticoagulant therapy, and the outcome is usually good [12].

On the other hand, mobile thrombus in the setting of PE is a marker of imminent and often fatal embolism. Reported mortality rates exceed 40% [12]. The presence of thrombus in the right heart is a contraindication for pulmonary angiography because of the risk of embolism.

Two major therapeutic options to consider for massive PE with right heart thrombus are thrombolysis and surgical thrombectomy. The detection of right atrial thrombus in the setting of PE increases the risk of death, and surgical thrombectomy should be considered [13]. A widely accepted indication for surgical thrombectomy is in patients with massive PE and hemodynamic instability, or if thrombolysis is either unsuccessful or contraindicated. In a retrospective analysis of 177 cases with right heart thromboembolism, 98% patients had PE, and the mortality associated with no treatment, using anti-coagulation only, surgical thrombectomy, and thrombolysis were 100.0%, 28.6%, 23.8%, and 11.3%, respectively. Overall mortality was reported to be 27.1% [14]. To date, there has been no randomized controlled trial comparing thrombolysis with surgical thrombectomy.

The appropriate treatment of submassive PE is not clear. The effects of thrombolysis on the prognosis of patients with acute submassive PE was studied in management strategies and the prognosis of pulmonary embolism-3 trial. The results showed that heparin combined alteplase could improve the clinical course of stable patients with acute submassive pulmonary embolism as well as prevent clinical deterioration, requiring an escalation of treatment during the hospital stay as compared to heparin plus placebo. During the study, only one fatal bleeding episode (in the heparin-plus-placebo group) was observed, with no incidence of hemorrhagic strokes. Low mortality was observed in both groups, and bleeding was not higher in the heparin-plus-alteplase group as compared to the heparin-plus-placebo group [15].

The optimal management of patients with paradoxical embolism from PFO remains controversial. Zuin et al. suggested that for patients with thrombophilia and PFO, the transcatheter closure of PFO, along with chronic oral anticoagulation, is recommended [3]. Data from long-term studies involving large patient populations for percutaneous PFO closure are still lacking. The practical and useful procedure for patients with paradoxical embolism from PFO is transcatheter closure due to its high success rate, low thromboembolic recurrence rate, and low incidence of complications [16]. However, randomized prospective studies are required to confirm the usefulness of this technique in the secondary prevention of cerebrovascular events. Also, the management of patients with acute PE with PFO is challenging, given an increased risk of a paradoxical embolism, which complicates the therapeutic approach. Two options that can be considered include catheter-directed thrombolysis with percutaneous PFO closure versus surgical embolectomy with the closure of PFO.

## Conclusions

Treatment of acute PE can be lifesaving, but the treatment approach is controversial, especially in the presence of complicating factors such as the patient’s co-morbidities, the clinical presentation or stability of the patient, and the adverse therapeutic effects of administered treatment. In this case, our elderly patient had multiple challenges in terms of therapeutic choices, and we strived to provide appropriate care for his complicated PE with due attention to not only current therapeutic guidelines but also a modification of approach for distinct challenges and the patient’s wishes.
